# Multisensor Systems by Electrochemical Nanowire Assembly for the Analysis of Aqueous Solutions

**DOI:** 10.3389/fchem.2018.00256

**Published:** 2018-06-29

**Authors:** Konstantin G. Nikolaev, Yury E. Ermolenko, Andreas Offenhäusser, Sergey S. Ermakov, Yulia G. Mourzina

**Affiliations:** ^1^Institute of Complex Systems ICS-8, Forschungszentrum Jülich GmbH, Jülich, Germany; ^2^JARA-FIT, Jülich, Germany; ^3^Institute of Chemistry, St. Petersburg State University, St. Petersburg, Russia

**Keywords:** electrochemical sensor, sensor array, metal nanowire assembly, multisensor system, non-enzymatic, glucose, ethanol, hydrogen peroxide

## Abstract

The development of electrochemical multisensor systems is driven by the need for fast, miniature, inexpensive, analytical devices, and advanced interdisciplinary based on both chemometric and (nano)material approaches. A multicomponent analysis of complex mixtures in environmental and technological monitoring, biological samples, and cell culture requires chip-based multisensor systems with high-stability sensors. In this paper, we describe the development, characterization, and applications of chip-based nanoelectrochemical sensor arrays prepared by the directed electrochemical nanowire assembly (DENA) of noble metals and metal alloys to analyze aqueous solutions. A synergic action of the electrode transducer function and electrocatalytic activity of the nanostructured surface toward analytes is achieved in the assembled metal nanowire (NW) sensors. Various sensor nanomaterials (Pd, Ni, Au, and their multicomponent compositions) can be electrochemically assembled on a single chip without employing multiple cycles of photolithography process to realize multi-analyte sensing applications as well as spatial resolution of sensor analysis by this single-chip multisensor system. For multi-analyte electrochemical sensing, individual amperometric signals of two or more nanowires can be acquired, making use of the specific electrocatalytic surface properties of the individual nanowire sensors of the array toward analytes. To demonstrate the application of a new electrochemical multisensor platform, Pd-Au, Pd-Ni, Pd, and Au NW electrode arrays on a single chip were employed for the non-enzymatic analysis of hydrogen peroxide, glucose, and ethanol. The analytes are determined at low absolute values of the detection potentials with linear concentration ranges of 1.0 × 10^−6^ − 1.0 × 10^−3^ M (H_2_O_2_), 1.5 × 10^−7^ − 2.0 × 10^−3^ M (glucose), and 0.7 × 10^−3^ − 3.0 × 10^−2^ M (ethanol), detection limits of 2 × 10^−7^ M (H_2_O_2_), 4 × 10^−8^ M (glucose), and 5.2 × 10^−4^ M (ethanol), and sensitivities of 18 μA M^−1^ (H_2_O_2_), 178 μA M^−1^ (glucose), and 28 μA M^−1^ (ethanol), respectively. The sensors demonstrate a high level of stability due to the non-enzymatic detection mode. Based on the DENA-assembled nanowire electrodes of a compositional diversity, we propose a novel single-chip electrochemical multisensor platform, which is promising for acquiring complex analytical signals for advanced data processing with chemometric techniques aimed at the development of electronic tongue-type multisensor systems for flexible multi-analyte monitoring and healthcare applications.

## Introduction

Electrochemical sensors and biosensors are widely used in clinical and pharmaceutical analysis, biomedical investigations, food quality assessments, as well as technological and environmental process monitoring due to their accuracy, selectivity, short response time, cost-effectiveness, applicability for multiparametric analysis, real-time *in situ* and *in vivo* measurements, and high spatial resolution achievable by miniaturization. Ongoing research in the field of electrochemical sensors and their operation principles leads to the development of new types of sensors and extends the range of possible applications. In particular, the need for flexible multi-analyte determinations in analyses of gas and liquid media has driven the research and development of new sensor materials, electrochemical mutisensor arrays, electronic nose- and electronic tongue-type systems (Persaud and Dodd, 1982; Di Natale et al., 1995; Vlasov et al., 1999, 2005, 2010; Maistrenko et al., 2011; Kirsanov et al., 2013; Peris and Escuder-Gilabert, 2013; Voitechovič et al., 2018).

For the multicomponent analysis of complex mixtures, electrochemical multisensor systems can be composed of sensors selective to individual analytes. In this case, individual sensors do not affect the accuracy of the determinations of other sensors of the multisensor system. The development of such multisensor arrays is required for miniaturization, lower costs, more reliable sensor analysis, and spatial resolution. In many cases, redox processes in amperometric and voltamperometric sensor applications on unmodified bare electrodes are hindered by the slow electrode kinetics and high overpotentials that must be applied for electrochemical conversion. As a consequence, the co-oxidation or reduction of many electroactive species present in natural samples can occur, causing unwanted interferences during detection which are difficult to distinguish. A wide range of materials and compounds with selective binding and/or electrocatalytic properties, which favor the thermodynamics and kinetics of specific redox processes, have been used to lower the absolute value of the response potential and improve the sensitivity and selectivity of these types of sensors (Yogeswaran and Chen, 2008; Budnikov et al., 2012; Thiyagarajan et al., 2014; Komkova et al., 2017). These include enzymes as biocatalysts (Heller and Feldman, 2008; Koposova et al., 2014, 2015; Nikolaev et al., 2015; Rocchitta et al., 2016; Quesada-González and Merkoçi, 2017; Bandodkar et al., 2018). However, there are some general problems associated with highly selective electrochemical sensors, where selectivity is achieved by enzymes such as the low stability of biosensors, restricted measurement conditions, the use of onerous immobilization procedures, and mediators, as well as poor compatibility with nanotechnology processing. Non-enzymatic sensors, which are proposed as an alternative to enzyme biosensors (Park et al., 2006; Toghill and Compton, 2010), often suffer from slow electrode kinetics, high overpotentials, and insufficient selectivity. The latter problem might possibly be resolved through the use of multisensor systems in combination with chemometric techniques. In this case, a multisensor system may include less selective or non-selective sensors with non-linear and multiparametric dependencies of the sensor signals on the component concentrations. A complex multiparametric signal of a multisensor system needs to be processed with chemometric techniques to obtain multiple analytical signals or non-parametric and non-quantitative information, as in the case of electronic nose and electronic tongue multisensor systems (Di Natale et al., 1995; Vlasov et al., 1999, 2010; Maistrenko et al., 2011; Panchuk et al., 2016). There are also some general problems with respect to electrochemical (bio) sensor analysis such as the need for new sensor materials and high-resolution sensor arrays. Therefore, multicomponent sensor analysis is advanced interdisciplinary based on chemometric and (nano)material approaches.

Electrochemical sensors based on metallic, carbonaceous, and composite nanomaterials help to advance the concept of non-enzymatic miniaturized electrochemical sensors (Chen et al., 2013, 2014; Guascito et al., 2013; Wang et al., 2016; Tee et al., 2017; Nikolaev et al., 2018) due to the electrocatalytic effects of surfaces and signal amplification techniques and could replace enzyme-based biosensors in various analytical applications. This leads to the improvement of sensor stability in fabrication and long-term use, cost-effectiveness, compatibility with nanotechnology, and could extend applications of sensors and multisensor systems. The development of electrochemical non-enzymatic sensors based on nanomaterials has been reviewed in a series of recent publications (Park et al., 2006; Chen et al., 2014; Jie et al., 2015; Zhang and Lieber, 2016; Gnana Kumar et al., 2017; Power et al., 2018).

Metal nanowires have become essential building blocks for the development of advanced, miniaturized non-enzymatic electrochemical sensors (Shaidarova and Budnikov, 2011; Chen et al., 2013, 2014; Koposova et al., 2013, 2015; Suib, 2013; Chen and Ostrom, 2015; Muratova et al., 2016). The improved electrocatalytic properties of the sensor metal nanomaterials in comparison with bulk materials are related to their high surface areas and energy, the preferential orientation of crystallographic planes, lattice defects at the surfaces, and the presence of pores, sharp edges, and nanoscale junctions. Different methods are available for the synthesis of metallic nanowires: chemical reduction from solutions, lithography technologies, the assembly by electromagnetic field forces, the template-based approach, CVD, laser deposition, etc. (Cheng et al., 2005; Nagashima et al., 2007; Kisner et al., 2011; Xing et al., 2012; Ji et al., 2013; Panov et al., 2017). Often, binary or more complex multicomponent systems demonstrate a higher (electro) catalytic activity due to synergic or electronic effects (Koper, 2004; Wang et al., 2008; Shaidarova and Budnikov, 2011; Guascito et al., 2013; Yang et al., 2013; Chen and Ostrom, 2015).

Recently, a method of the directed electrochemical nanowire assembly (DENA) was proposed for metal nanowires (Cheng et al., 2005, 2011; Talukdar et al., 2006; Ozturk et al., 2007a,b; Kawasaki and Arnold, 2011; Flanders et al., 2012; Ji et al., 2013; Zhang et al., 2013; Yi et al., 2014; Nikolaev et al., 2017). The method is based on the directional growth of metal nanowires and nanodendrites under the action of an AC voltage of high-frequency and a DC offset voltage applied between a pair of pre-structured electrodes. Using this method, different metal nanowire compositions can be electrodeposited on a chip and connected to the external circuitry in a single step. Important advantages of DENA, such as application at room temperature and atmosphere, spatial resolution, fast rates of the directional electrodeposition of metal nanostructures, and low costs have yet to be fully explored for electrochemical sensors and multisensor systems. In our recent work, we explore the DENA approach for a novel class of electrochemical multisensor systems in electrolyte solutions (Nikolaev et al., 2017, 2018). A synergic action of the electrode transducer function of the assembled Pd-Au nanowires and the electrochemical activity of the nanowire surface toward hydrogen peroxide reduction is achieved in the proposed multisensor system (Nikolaev et al., 2018). The detection of hydrogen peroxide in cardiomyocyte-like HL-1 cells using a non-enzymatic Pd-Au nanowire multisensor array prepared by DENA was also demonstrated (Nikolaev et al., 2018). The DENA method has been applied to create nanostructures of platinum (Kawasaki and Arnold, 2011), gold (Ozturk et al., 2007a; Nikolaev et al., 2017), palladium (Nikolaev et al., 2018), as well as Au-Pt (Cheng et al., 2011) and Au-Ag (Ji et al., 2014) compositions. Such materials can enable the detection of a number of analytes (Koposova et al., 2014; Ermakov et al., 2016; Nikolaev et al., 2017).

In this paper, we describe the development, characterization, and application of the chip-based nanoelectrochemical multisensor platform prepared by DENA for the analysis of liquid media. A single-chip multisensor system is composed of an array of Pd-Au, Pd-Ni, Pd, and Au NW electrodes prepared without employing multiple cycles of photolithography process to realize a multiplicity of the NW sensor compositions on a single chip. Individual amperometric signals of two or more nanowires can be acquired, making use of the specific electrocatalytic surface properties of the individual nanowire sensors of the array for the electrochemical detection. The multisensor system was employed for the non-enzymatic analysis of hydrogen peroxide, glucose, and ethanol. The proposed nanoelectrochemical multisensor platform is promising for acquiring complex analytical signals for advanced data processing with chemometric techniques for flexible multi-analyte monitoring and healthcare applications.

## Materials and methods

### Chemicals

Potassium tetrachloropalladate (II), palladium (II) chloride, gold (III) chloride trihydrate, nickel (II) chloride, disodium phosphate, hydrogen peroxide (≥30%), HEPES (4-(2-hydroxyethyl)-1-piperazineethanesulfonic acid), sodium dihydrogen phosphate, propanol, acetone, and other chemicals were obtained from Sigma-Aldrich. The substances were of analytical grade purity and used without further purification. The photoresists and developer for the photolithography were obtained from MicroChem Corp. and MicroChemicals GmbH. Solutions of analytes were prepared directly before measurements. All solutions were prepared using distilled water.

### Electrode synthesis by directed electrochemical nanowire assembly

**Figure 1:**
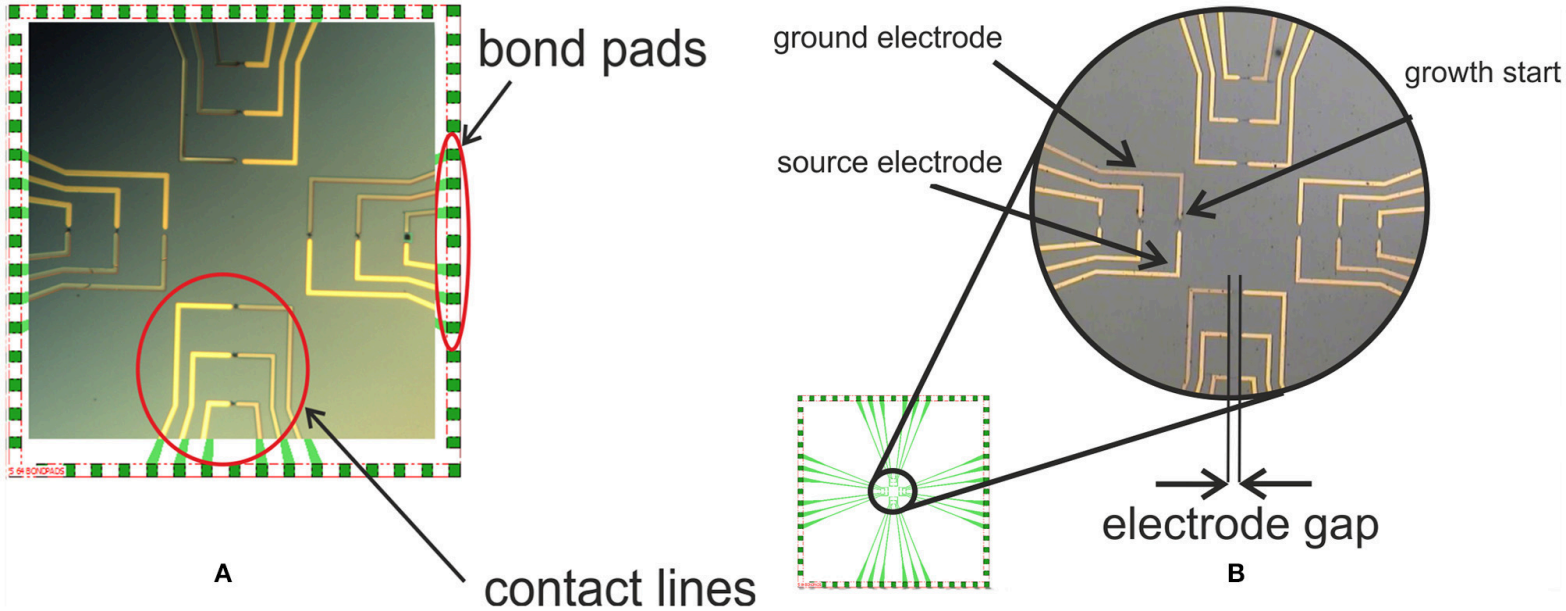
Design of a single 13 × 13 mm chip **(A)**. Source and ground electrodes setup, growth initiation site, and electrode gap **(B)**.

The growth microelectrodes (source and ground electrodes), Figure 1, for the electrodeposition of the nanowires of various compositions by DENA were structured on the substrates and produced using thin-film technologies in an ISO 5 cleanroom as described in detail (Nikolaev et al., 2018). Briefly, wafers of single-crystal boron-doped n-Si with (111) surface orientation were employed as substrates for fabricating electrochemical sensors. The silicon wafers were oxidized to produce a silicon dioxide layer of 1000 nm thickness using a Tempress oxidation furnace. These Si/SiO_2_ wafers were further used as carriers for the deposition of the growth microelectrodes by photolithography and lift-off processes.

After dehydration at 180°C for 20 min, the Si/SiO_2_ wafers were coated with a LOR 3B photoresist to produce a layer of 5 μm, baked for 5 min, and subsequently coated with an AZ nLOF 2020 photoresist to produce a layer of 2 μm. A photoresist stack was used instead of a single photoresist layer to achieve a better control over the geometry of the growth microelectrodes and contact lines. After pre-baking at 115°C for 90 s, the Si/SiO_2_ wafers with a photoresist stack were exposed at 325 watt using a photolithography mask at Mask Süss MA-6 (Hg-vapor lamp 350 W). Exposure time was optimized as 1.4 s. After exposure, the wafers were post-exposure baked at 115°C for 90 s. The wafers with an exposed photoresist stack were subsequently developed by AZ® 326 (MIF, 2.38% TMAH in H_2_O) for 1 min to produce structured photoresist layers for subsequent metallization and lift-off processes.

Thin metal layers of titanium for adhesion (10 nm) and gold (100 nm) were deposited on the Si/SiO_2_ wafers with pre-structured photoresist layers by means of an electron beam evaporation using a Pfeiffer PLS 500 equipment. Afterwards, the wafers were lifted off using acetone to remove sacrificial photoresist polymer layers. The wafers were then cleaned in isopropanol and distilled water.

Subsequently, DENA of nanowires and nanodendrites was performed on the Si/SiO_2_ wafers with metal bond pads, contact lines, and growth electrodes prepared by photolithography and lift-off processes as described above. The nanowires and nanodendrites of various metal and bimetallic compositions were assembled between growth electrodes using an Agilent Trueform Series Waveform Generator 33600. The optical microscope Leica DMLB was used for fixing and observation of DENA.

### Analysis by scanning electron microscopy and energy-dispersive X-ray spectroscopy

The nanoelectrodes were characterized by scanning electron microscopy using a Magellan™ XHR SEM equipped with an energy-dispersive X-ray spectroscopy (EDX) detector system, and a Magellan™ XHR SEM equipped with a focused ion beam (FIB) setup.

### Electrochemical measurements

To be used in aqueous solutions, the contact lines and growth electrodes of the nanowire electrode arrays prepared by DENA need to be protected by an isolation layer. An isolation layer of polyimide was produced by means of an aligned photolithography procedure. The channels with arrays of the assembled nanowire electrodes were left free from the isolation polyimide layer for contact with aqueous solutions. Additionally, the bond pads were left open for making electrical contacts to the external circuit. The wafers were diced into 13 × 13 mm chips. A glass ring with a radius of about 8 mm was attached on top of each chip to make an electrochemical cell compartment and accommodate the electrolyte solution for electrochemical experiments using polydimethylsiloxane (PDMS, 10:1, Sylgard). The bond pads were left outside the glass ring.

Electrochemical experiments were performed using a three-electrode electrochemical cell placed in a dark Faraday cage and controlled by a potentiostat-galvanostat AUTOLAB (PGSTAT 302, The Netherlands). The electrochemical cell was made of a coiled platinum auxiliary electrode, an Ag/AgCl reference electrode (3 M KCl, DRIREF-450, World Precision Instruments), and the desired working electrode. The electrochemical cell was covered with a PDMS top cover, which incorporated apertures for the electrodes and argon supply. The solutions and electrochemical cell were purged with argon to remove oxygen and kept under argon atmosphere during the experiments. All experiments were at 22 ± 1°C. A detection limit of sensors was estimated using a signal to noise (S/N) ratio of 3.

## Results and discussion

### Growth and characterization of the nanoelectrochemical sensors

In a typical experiment on the nanowire assembly, the growth electrodes, Figure 1, were connected to the AC/DC voltage generator via bond pads and a small volume of the metal salt solution of about 5 μl was positioned across the electrode gap. A square wave potential of the defined frequency and a DC voltage offset were applied across the electrode gap. Experimentally optimized growth parameters and solution compositions are shown in Table 1. DENA was detected in real time by optical microscopy (a video of the nanowire assembly process is available in Supporting Information).

**Table 1:** The experimentally optimized parameters for the directed electrochemical nanowire electrode assembly.

**Composition**	**Growth parameters**	
	**AC, DC parameters**	**Solution composition**
Au	45 MHz 18 V_pp_, 1 V_DC_	1 × 10^−2^ M HAuCl_4_ in H_2_O
Pd	45 MHz 17 V_pp_, 1.5 V_DC_	1 × 10^−2^ M K_2_PdCl_4_ in H_2_O
Pd-Au	40 MHz 17 V_pp_, 1.5 V_DC_	5 × 10^−3^ M K_2_PdCl_4_, 5 × 10^−3^ M HAuCl_4_ in water or PBS
Pd-Ni	38 MHz 15 V_pp_, 2.5 V_DC_	2.5 × 10^−3^ M PdCl_2_, 7.5 × 10^−3^ M NiCl_2_ in 0. 1 M HEPES buffer

**Figure 2:**
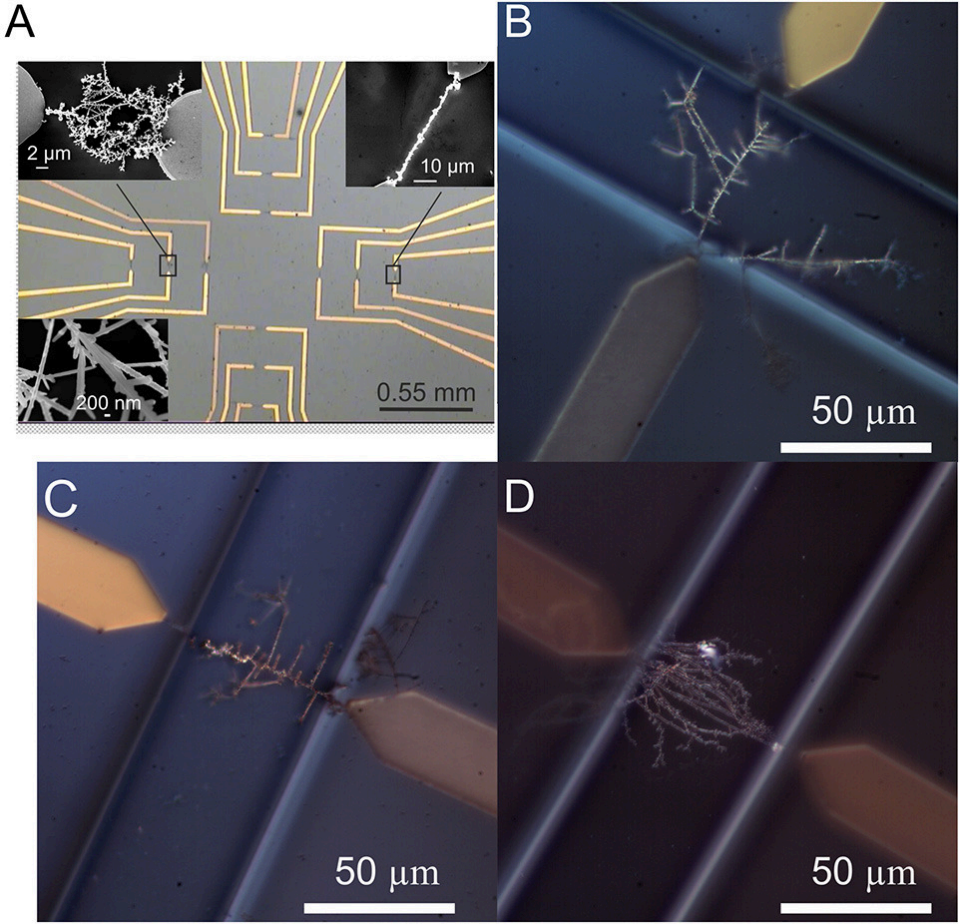
**(A)** Optical microscopy image of a 13 × 13 mm chip with contact paths and growth electrodes structured by photolithography, and the nanowire electrodes assembled by DENA (contact pads are not shown on the image). Inserts in **(A)** show SEM images of Pd-Au (up left), Pd (up right), and the structural features of an Au (below) nanowire electrodes prepared by DENA. Optical microscopy images of Au **(B)**, Pd **(C)**, and Pd-Au **(D)** nanowire, and nanodendrite electrodes prepared by DENA after isolation with polyimide polymer.

**Figure 3:**
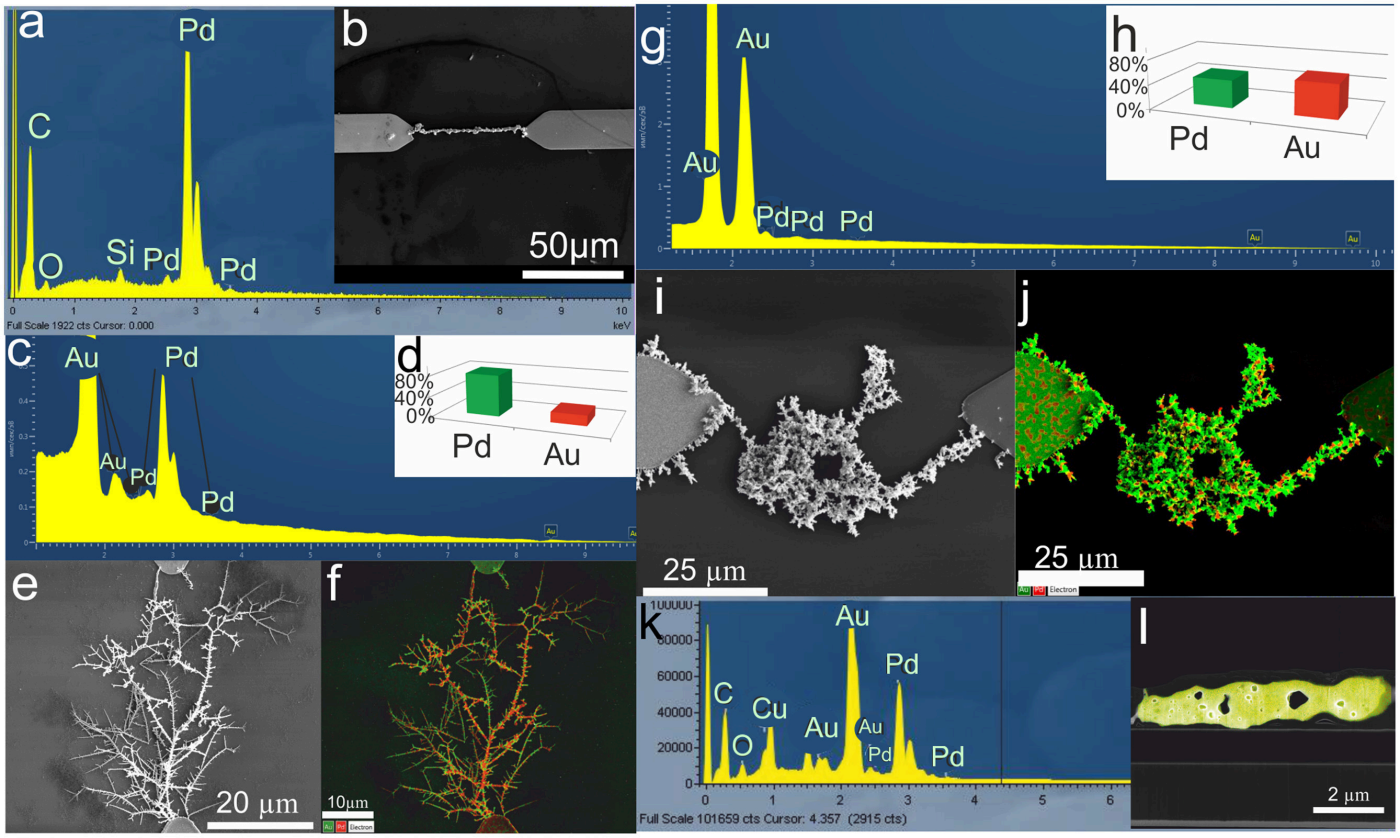
**(a)** EDX spectrum of a Pd NW electrode, **(b)** SEM micrograph of the Pd NW electrode shown in **(a)**. The Pd NW electrode was synthesized by electrodeposition from a Pd (ac)_2_ solution. **(c)** EDX spectrum of a Pd-Au nanodendrite electrode and **(d)** histogram of the Pd and Au element content in the Pd-Au nanodendrite shown in **(e)** the SEM micrograph. **(f)** SEM micrograph of the same Pd-Au nanodendrite electrode with elemental mapping (Pd-green, Au-red). The Pd-Au nanodendrite electrode was electrochemically assembled in a 5 × 10^−3^ M HAuCl_4_ and 5 × 10^−3^ M K_2_PdCl_4_ water solution at 45 MHz, 17 V_pp_, and 1.5 V DC offset. **(g)** EDX spectrum of a Pd-Au nanodendrite electrode and **(h)** histogram of the Pd and Au element content in the Pd-Au nanodendrite electrode shown in **(i)** the SEM micrograph. **(j)** EDX elemental mapping of the same Pd-Au nanodendrite electrode (Pd-green, Au-red). **(k)** EDX spectrum of the lamella (cross section) of the Pd-Au nanodendrite electrode. **(l)** EDX elemental mapping of the same lamella of the Pd-Au nanodendrite electrode. The structure was synthesized by electrodeposition from 5 × 10^−3^ M HAuCl_4_ and 5 × 10^−3^ M PdCl_2_ dissolved in PBS, pH 8, at 38 MHz, 17 V_pp_, and 1.5 V DC offset. Reprinted by permission from: Springer, J. Solid State Electrochemistry, Bimetallic nanowire sensors for extracellular electrochemical hydrogen peroxide detection in HL-1 cell culture, Konstantin G. Nikolaev, Vanessa Maybeck, Elmar Neumann, Sergey S. Ermakov, Yury E. Ermolenko, Andreas Offenhäusser, Yulia G. Mourzina © (2017), advance online publication, 28.11.2017 (doi: 10.1007/s10008-017-3829-3).

**Figure 4:**
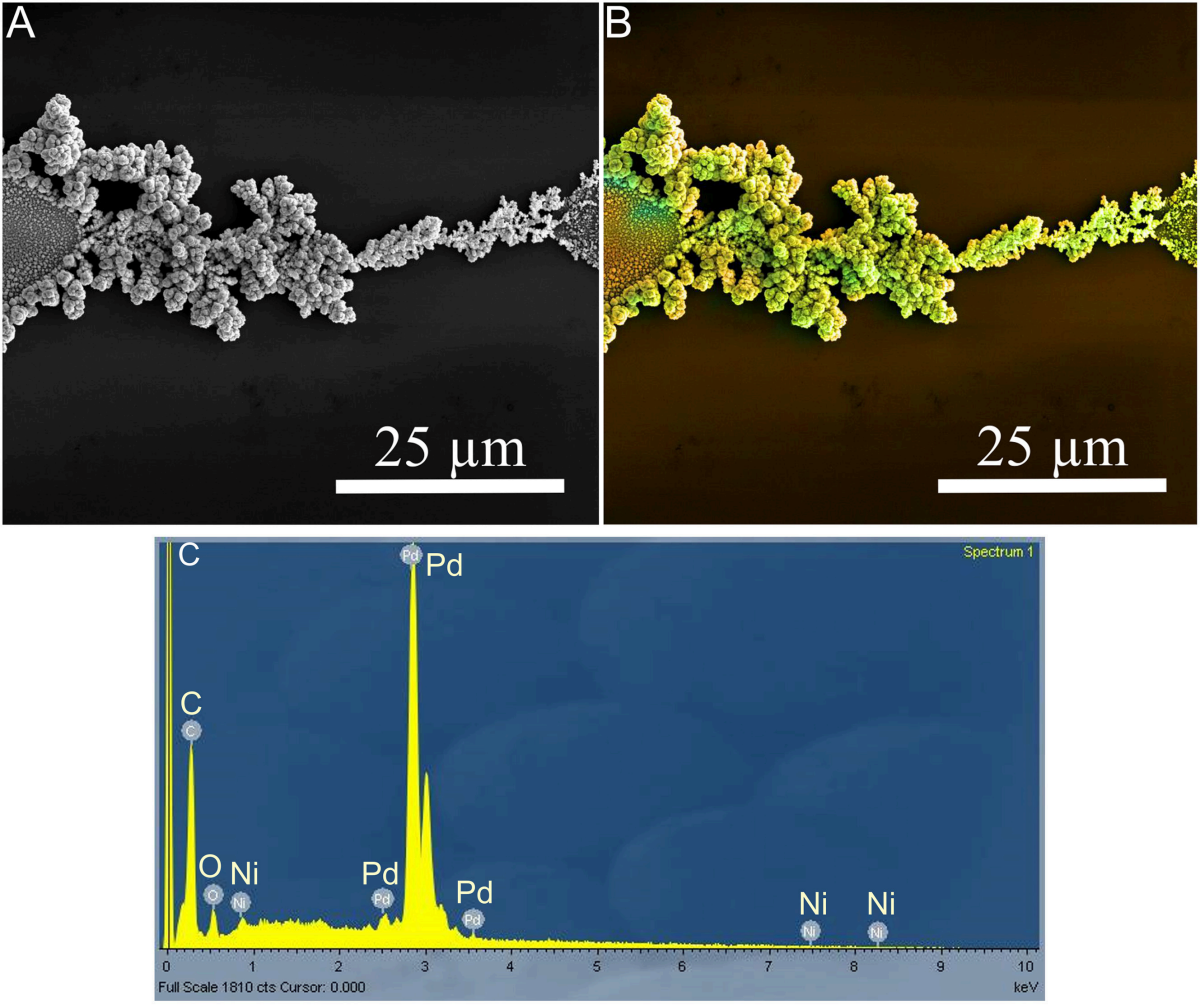
**(A)** SEM micrograph, **(B)** EDX map (Pd is red, Ni is green), and **(C)** EDX spectrum of the Pd-Ni nanodendrite electrode. This structure was synthesized at 38 MHz, 15 V_pp_, and 2.5 V DC offset.

Nanowire shape and compositions vary with the composition of the electrodeposition solution, electrode gap, and AC/DC parameters, as shown for example in Figures 2–**4**. The nanodendrite electrodes with an overall diameter from 50 nm to several micrometers were obtained by DENA in our studies. The details of the growth mechanism were discussed in Bockris and Despic (1970), Cheng et al. (2005), Ozturk et al. (2007a,b), Kawasaki and Arnold (2011), Ji et al. (2013), and Nikolaev et al. (2018). For the multisensor system, several types of electrodes, i.e., Pd, Au, Pd-Au, and Pd-Ni, were grown on a single chip.

Figure 3 shows an example of a Pd electrode, Figures 3A,B, and two examples of Pd-Au nanodendrite electrodes. A Pd-Au nanodendrite electrode in Figures 3G–L was obtained by deposition from 5 × 10^−3^ M HAuCl_4_ and 5 × 10^−3^ M PdCl_2_ in a 0.05 M phosphate buffer solution (PBS) of pH 8 at 38 MHz, 17 V_pp_, and 1.5 V DC offset. According to the EDX elemental mapping and spectrum of the nanowire composition, gold and palladium were homogeneously distributed throughout the cross-section and length of the Pd-Au nanodendrite electrode. The element content was found to constitute 55.5 and 44.5% for Au and Pd, respectively, while Pd accounted for about 60% in a Pd-Au nanodendrite electrode in Figures 3c–f in case of deposition from water solution. The nanodendrite electrodes have typically an electrochemically active surface area of thousands of μm^2^ (Nikolaev et al., 2018).

The co-deposition of Pd and Ni was carried out at 38 MHz, 15 V_pp_, and 2.5 V DC offset in a solution of 2.5 × 10^−3^ M K_2_PdCl_4_ and 7.5 × 10^−3^ M NiCl_2_ positioned across a 50 μm electrode gap, as shown in Figure 4. The chemical composition of as-prepared Pd-Ni electrode was determined using an EDX analysis. Figures 4C,B show the corresponding EDX spectrum and mapping of Pd (red) and Ni (green), respectively.

### Electroanalytical measurements

#### Detection of glucose

**Figure 5:**
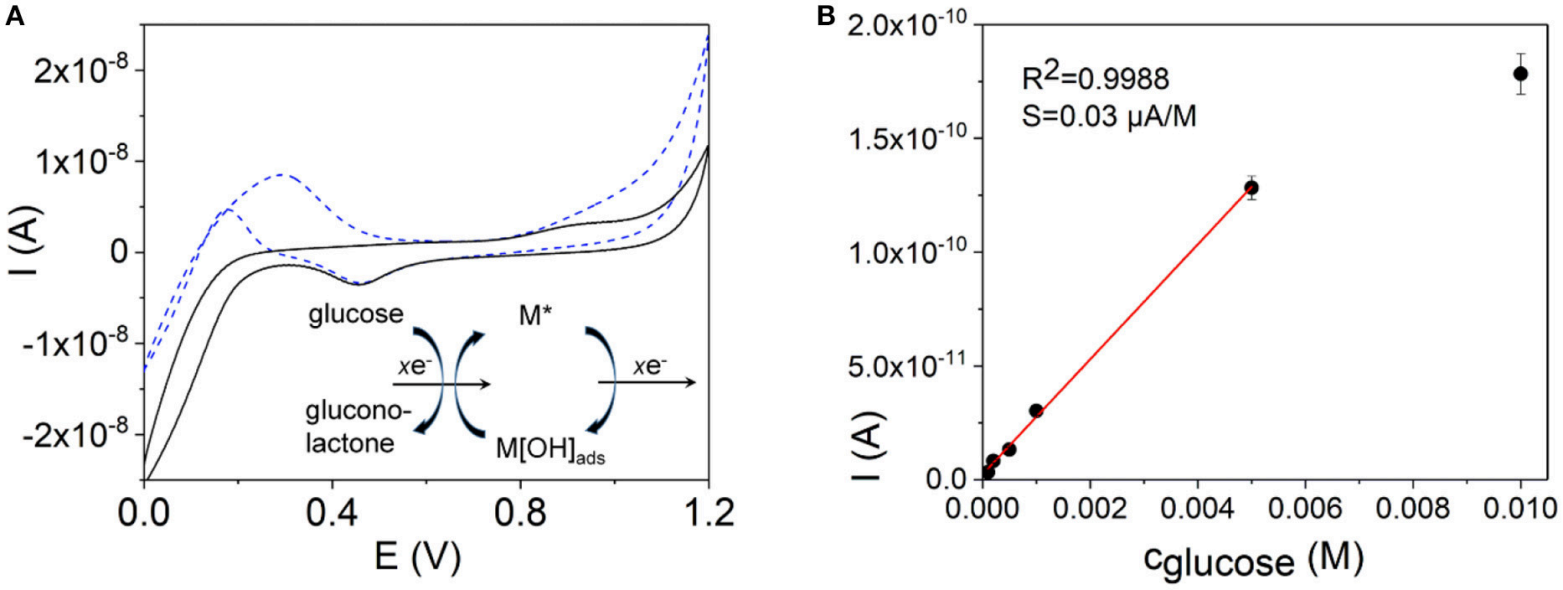
**(A)** Cyclic voltammograms of an Au NW electrode in a 0.1 M PBS, pH 7.2, (black line) and in a 1 × 10^−2^ M solution of glucose in PBS (blue dashed line). Insert in **(A)** shows a scheme of the oxidation and reduction processes at the metal electrode surface during oxidation of glucose. **(B)** A calibration curve as a current dependence on the concentration of glucose. The calibration curve was obtained by means of amperometry at E_det_ = +0.35 V (vs. Ag/AgCl) using an Au NW electrode prepared by DENA as a working electrode.

**Figure 6:**
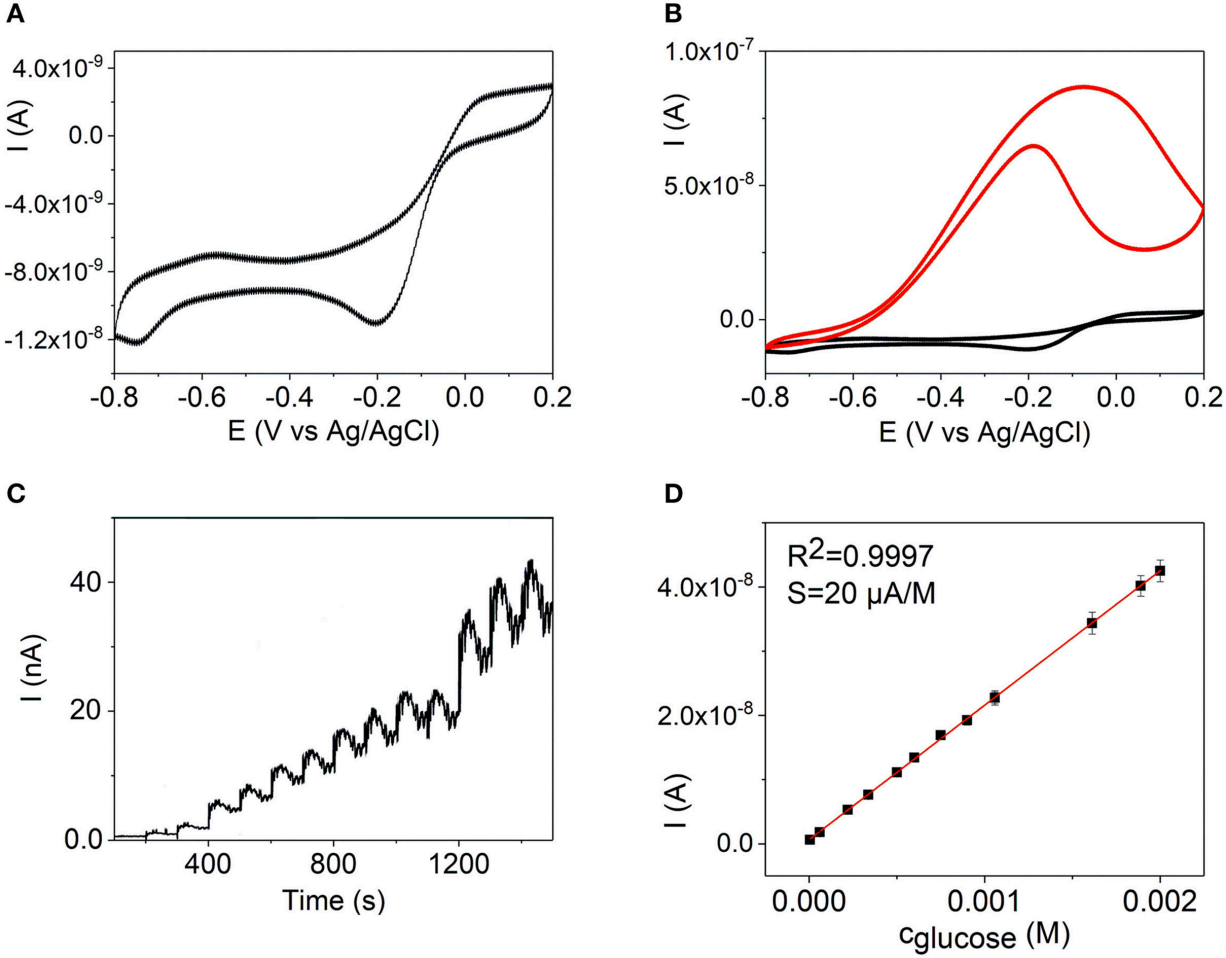
Cyclic voltammograms of a Pd NW electrode in 0.1 M KOH (black line) **(A)** and with addition of 2.0 × 10^−3^ M of glucose (red line) **(B)**. **(C)** Amperometric detection of glucose on a Pd NW electrode in 0.1 M KOH at −0.15 V with additions of glucose up to 2.0·10^−3^ M. **(D)** Calibration curve as a dependence of the oxidation current on the concentration of glucose at −0.15 V at a Pd NW electrode, 0.1 M KOH.

**Figure 7:**
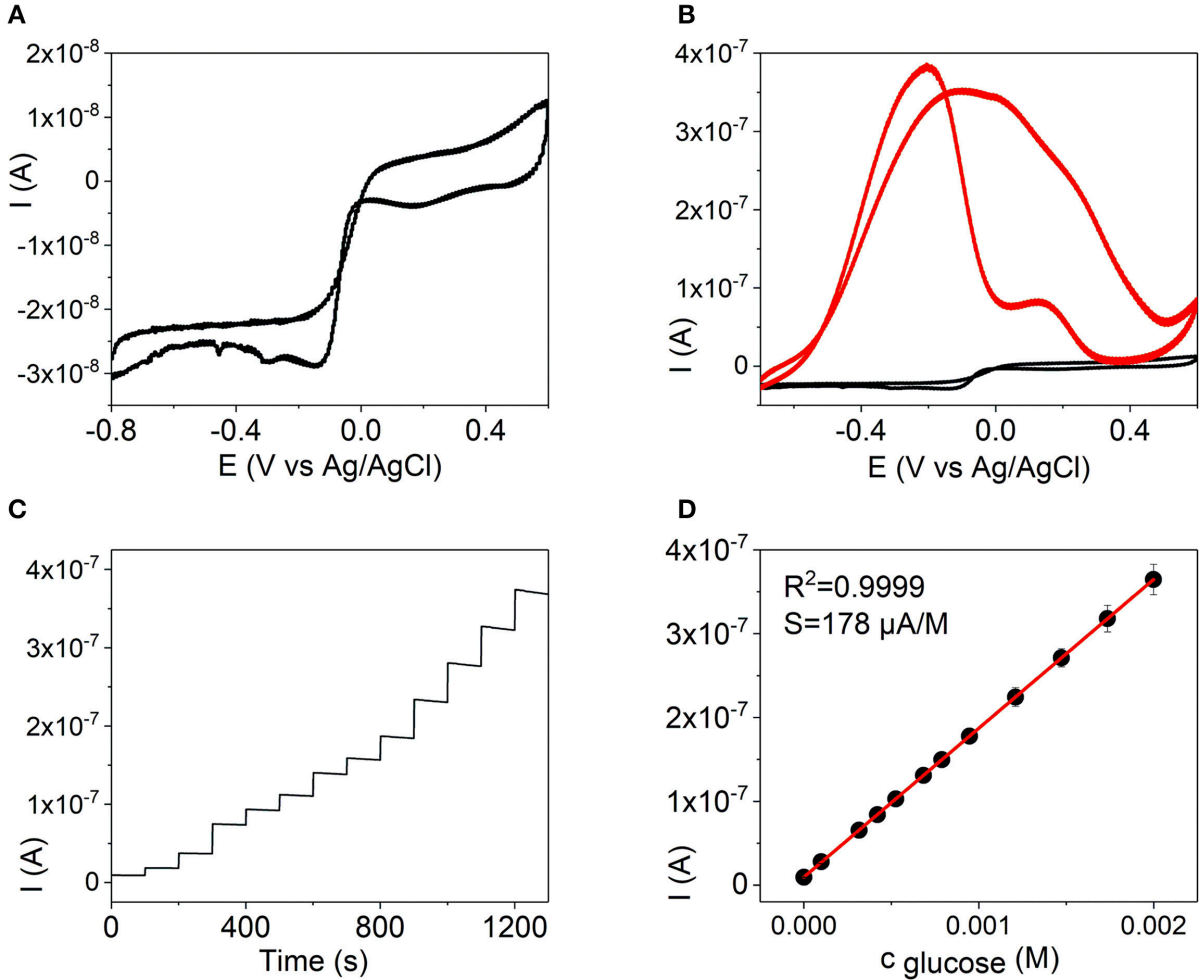
Cyclic voltammograms of a Pd-Ni NW electrode in 0.1 M KOH **(A)** and in 0.1 M KOH (black line) with addition of 2.0 × 10^−3^ M glucose (red line) **(B)**. **(C)** Amperometric detection of glucose on a Pd-Ni NW in 0.1 M KOH at −0.15 V with additions of glucose up to 2.0 × 10^−3^ M. **(D)** Calibration curve as a current dependence on the concentration of glucose at −0.15 V at a Pd-Ni NW electrode, 0.1 M KOH.

The metal nanodendrite electrodes prepared by DENA were further characterized by cyclic voltammetry in 0.1 M PBS (pH 7.2) or 0.1 M KOH, Figures 5A, 6A, 7A. In a number of cases, the current-voltage curves indicated that the connections between the nanowire electrodes and growth electrodes were destroyed during the isolation procedure. Therefore, these electrodes were not used for further experiments.

The electrochemical activities of different nanodendrite electrode surfaces prepared by DENA toward glucose oxidation were studied to evaluate the feasibility of their application for glucose sensing, Figures 5–7. Non-enzymatic glucose electrochemical sensors have been presented and discussed in a number of recent reviews and articles (Toghill and Compton, 2010; Chen and Ostrom, 2015; Jin et al., 2015; Pourbeyram and Mehdizadeh, 2016; Liu et al., 2017; Quesada-González and Merkoçi, 2017; Shabnam et al., 2017; Barragan et al., 2018). The electrochemical measurements for the Au NW electrode were performed in a 0.1 M PBS at pH 7.2, Figure 5. Cyclic voltammograms of the electrodes were recorded in a potential range of 0–1.2 V (vs. Ag/AgCl), Figure 5A. As shown in Figure 5A, the CV of the Au NW electrode in the absence of glucose displays the characteristic features of a gold electrode surface. The gold cathodic peak potential after anodic oxidation in the positive (anodic) scan was at about +0.45 V for the Au nanodendrite electrode, Figure 5A (black line). Cyclic voltammogram in a 10^−2^ M solution of glucose displays an anodic current due to oxidation of glucose in an anodic (forward) scan at about +0.29 V and an electrocatalytic oxidation current at about +0.17 V in a cathodic (reverse) scan, Figure 5A (blue dashed line). In the reverse (cathodic) potential sweeping, the reduction of the oxidized gold surface occurs, meaning that the active sites on the electrode surface are regenerated and accessible for the glucose oxidation. According to this, anodic current of the glucose oxidation with a new peak at about +0.17 V is observed in a reverse scan, Figure 5A (blue dashed line). The Au NW non-enzymatic sensor demonstrated a linear concentration range of 1 × 10^−4^ − 5 × 10^−3^ M with a sensitivity of 0.03 μA M^−1^ and a lower limit of detection of 3.3 × 10^−5^ M glucose. Selectivity of the Au NW sensor in the presence of fructose, sucrose, and ascorbic acid is shown in Figure S1.

Mechanisms of non-enzymatic electrocatalysis of glucose oxidation on metal electrode surfaces has generated much interest over the years for applications in glucose sensors and glucose-oxygen biofuel cells (Nikolaeva et al., 1983; Vassilyev et al., 1985; Makovos and Liu, 1986; Burke, 1994; Heller and Feldman, 2008; Pasta et al., 2010; Toghill and Compton, 2010). However, the electrocatalytic behavior of gold electrodes in this process is complex and although many studies were published and a series of mechanisms were proposed, the mechanism of the glucose oxidation process remains uncertain. The processes of electrocatalytic transformations on electrode surfaces generally proceed via the adsorption of the analyte to the surface of the electrode via suitable bonds, which should be formed or broken during catalytic process (Pletcher, 1984) and involve the d-electrons and d-orbitals of the metal electrodes surface. Most notably is that electrocatalytic oxidation of glucose is favored on the oxidized metal surfaces of surface oxides or hydroxides and it was noted that electrooxidation of a number of organic molecules including glucose coincided with the OH_ads_ formation (Toghill and Compton, 2010). The concept that the oxide mediator species were involved in the electrocatalytic oxidation processes at electrodes was postulated earlier by Bagotzky and Vassilyev (1967) for the process of methanol oxidation on Pt. Burke (1994) discussed the premonolyer incipient hydrous oxide layer formed in a premonolayer oxidation step on the metal electrode surface and its role on the electrocatalytic processes at noble metal electrode/aqueous solution interface. The author introduced the “Incipient Hydrous Oxide Adatom Mediator” theory, where OH_ads_ oxy-species on noble metal electrode surfaces might act as mediators of many electrocatalytic processes. It is thus believed that incipient hydrous gold oxide, which is formed by chemisorption of OH^−^ to the surface of a gold electrode mediates electrocatalytic processes at a gold electrode. This hydrous gold oxide premonolayer may favor the process of the removal of the hemiacetalic hydrogen atom from the glucose molecule (Park et al., 2006) and mediates oxidation of the adsorbed species. Therefore electrocatalytic effect is more pronounced at higher pH, since the reversible adsorption of oxygen in the form of OH_ads_ on the gold electrode surfaces is negligible in acidic media (Vassilyev et al., 1985). Therefore, non-enzymatic glucose sensors based on metallic nanostructures are mostly used in alkaline solutions.

Nikolaeva et al. (1983) and Vassilyev et al. (1985) proposed a mechanism, in which an electrochemically formed gold oxide on the gold electrode surface possessed a catalytic effect on the process of glucose electrooxidation. It is thus supposed that a chemisorption of glucose at the hydrous gold oxide takes place and the adsorbed glucose is then oxidized by the adsorbed hydroxide anions, like it is schematically shown in Equations (1,2), and a scheme in Figure 5A:

(1) $$\begin{array}{l} \text{Au}\left[\text{O}{\text{H}}_{\text{ads}}\right]+\text{glucose}\to \text{Au}+\text{gluconolactone or gluconic acid} \end{array}$$

or

(2) $$\begin{array}{l} \text{A}{\text{u}}_{\text{n}}{\text{O}}_{\text{m}}\;+\text{ glucose}\to \text{A}{\text{u}}_{\text{n}}{\text{O}}_{\text{m}-1}+\text{gluconolactone or gluconic acid} \end{array}$$

The mechanism was further elaborated by Makovos and Liu (1986), which observed an anodic current during the cathodic scan in cyclic voltammetry and the dependence of the peak current values on the concentration of glucose. A common final step in Nikolaeva et al. (1983) and Makovos and Liu (1986) was a rapid electrochemical regeneration of the gold surface oxy-species as illustrated in Equations (3–5), and scheme in Figure 5:

(3) $$\begin{array}{l} \text{O}{\text{H}}^{-}\to \text{O}{\text{H}}_{\text{ads}}+{\text{e}}^{-} \end{array}$$

(4) $$\begin{array}{l} {\text{H}}_{2}\text{O}\to \text{O}{\text{H}}_{\text{ads}}++{\text{H}}^{+}+{\text{e}}^{-} \end{array}$$

or

(5) $$\begin{array}{l} \text{A}{\text{u}}_{\text{n}}{\text{O}}_{\text{m}-1}+2\text{O}{\text{H}}^{-}\to \text{A}{\text{u}}_{\text{n}}{\text{O}}_{\text{m}}+{\text{H}}_{2}\text{O}+2{\text{e}}^{-} \end{array}$$

The mechanism of direct electrocatalytic glucose oxidation on Pd electrocatalysts was reported and is summarized in Equations (6–8) (Vassilyev et al., 1985; Becerik and Kadirgan, 1992; Cai et al., 2013; Chen et al., 2015):

(6) $$\begin{array}{l} \text{Pd}+\text{glucose}\to \text{Pd}-{\text{H}}_{\text{ads}}+\text{intermediates} \end{array}$$

(7) $$\begin{array}{l} \text{Pd}+\text{O}{\text{H}}^{-}\to \text{Pd}\left[\text{O}{\text{H}}_{\text{ads}}\right]+{\text{e}}^{-} \end{array}$$

(8) $$\begin{array}{l} \text{PdO}{\text{H}}_{\text{ads}}+\text{glucose or intermediates}\to \text{Pd}\\ +\text{gluconolactone or gluconic acid} \end{array}$$

In a reverse (cathodic) scan, Figure 6B, the reduction of Pd oxide and reformation of active hydrous oxide species, Pd(OH)_x_, occurs after about 0.01 V in alkaline medium. The regenerated active sites of Pd hydroxide species carry out glucose oxidation reaction again and the anodic peak of glucose re-occurs.

Nickel electrodes were also investigated as catalysts for the electrooxidation of organic substances in alkaline medium, where the oxidized component of the Ni(OH)_2_/NiOOH redox couple is a catalytic component (Fleischmann et al., 1971; Toghill and Compton, 2010). It is supposed that the removal of the hemiacetalic hydrogen atom is the rate determining step of the process of glucose electrooxidation at nickel oxyhydroxide. Thus, the oxidation of glucose to gluconolactone in an alkaline solution is catalyzed by the Ni(II/III) redox couple of the electrode surface in accordance with reactions (9–10) (Toghill and Compton, 2010; Li et al., 2014):

(9) $$\begin{array}{l} \text{Ni}{\left(\text{OH}\right)}_{2}+\text{O}{\text{H}}^{-}\to \text{NiO}\left(\text{OH}\right)+{\text{H}}_{2}\text{O}+{\text{e}}^{-} \end{array}$$

(10) $$\begin{array}{l} \text{NiO}\left(\text{OH}\right)+\text{glucose}+\text{O}{\text{H}}^{-}\to \text{Ni}{\left(\text{OH}\right)}_{2}\\ +\text{gluconolactone}+{\text{H}}_{2}\text{O}+2{\text{e}}^{-} \end{array}$$

Non-enzymatic electrodes based on electrocatalysis, which rely on bimetallic systems, may offer electrodes, in which the catalytic and electronic benefits of the components synergistically combine to reach particular electronic and catalytic properties (Vassilyev et al., 1985; Wang et al., 2008; Toghill and Compton, 2010; Shaidarova and Budnikov, 2011; Zhang et al., 2011; Si et al., 2013; Yang et al., 2013; Chen and Ostrom, 2015). A high surface area of the nanodendrite electrodes provides active sites for the electrocatalytic reaction. Moreover, the dendritic metals have many nanostructured features such as sharp edges and nanoscale junctions rendering them a high activity.

Figures 6A,B, 7A,B show the CV curves of Pd and Pd-Ni NW electrodes in the absence and presence of 2.0 × 10^−3^ M glucose in 0.1 mol L^−1^ KOH. In order to evaluate the sensitivity of the sensors to glucose, amperometric responses of the Au, Pd, and Pd-Ni nanodendrite electrodes to the changes of the glucose concentration were studied. The amperometric measurements were made in a constantly stirred 0.1 M solution of potassium hydroxide with successive additions of glucose every 100 s, Figures 6C, 7C. As can be seen in Figures 6D, 7D, well-defined amperometric currents were proportional to the concentration of glucose in a concentration range of 5.0 × 10^−6^ to 2.0 × 10^−3^ M for the Pd NW electrode and 1.5 × 10^−7^ to 2.0 × 10^−3^ M for the Pd-Ni NW electrode, respectively.

Series of CVs experiments were performed to evaluate linear concentration ranges, LODs, sensitivities, and regression coefficients. The experiments were performed in 0.1 M KOH for Pd, Figure 6, and Pd-Ni, Figure 7, electrodes, and in 0.1 M PBS for the Au NW electrode, Figure 5. Table 2 summarizes the analytical characteristics of the Au, Pd, and Pd-Ni nanodendrite electrodes assembled by DENA in our studies for the detection of glucose. As shown in Table 3, the calibration graphs were linear in concentration ranges 1.0 × 10^−4^ − 5.0 × 10^−3^ M for the Au NW electrode, 5.0 × 10^−6^ − 2.0 × 10^−3^ M for the Pd electrode, and 1.5 × 10^−7^ − 2.0 × 10^−3^ M for the Pd-Ni nanodendrite electrode. The LODs were 3.3 × 10^−5^ M, 1.3 × 10^−6^ M, and 4.0 × 10^−8^ M for Au, Pd, and Pd-Ni electrodes, respectively (*S*/*N* = 3). Regression coefficients for all electrodes compositions were not < 0.9988. Electrodes sensitivities in linear concentration ranges were found to be 0.03, 20, and 178 μA M^−1^ for Au-, Pd-, and Pd-Ni nanodendrite electrodes, respectively.

**Table 2:** Analytical characteristics of the nanodendrite electrodes prepared by DENA for the detection of glucose.

**Electrode**	**E_det_, V**	**Linear range, M**	**LOD, M**	**Sensitivity, μA M^−1^**
Au	0.35V(PBS)	1.0 × 10^−4^ − 5.0 × 10^−3^	3.3 × 10^−5^	0.03
Pd	−0.15(KOH)	5.0 × 10^−6^ − 2.0 × 10^−3^	1.3 × 10^−6^	20
Pd-Ni	−0.15(KOH)	1.5 × 10^−7^ − 2.0 × 10^−3^	4.0 × 10^−8^	178

**Table 3:** Analytical characteristics of the ethanol detection with the nanodendrite electrodes prepared by DENA.

**Electrode**	**E_det_, V**	**Linear range, M**	**LOD, M**	**Sensitivity, μA M^−1^**
Pd	−0.3	7.0 × 10^−4^ − 3.0 × 10^−2^	2.2 × 10^−4^	1.5
Pd-Ni	−0.25	7.0 × 10^−4^ − 3.0 × 10^−2^	5.2 × 10^−4^	28

One can see from Table 2 that although all three compositions demonstrate sensor properties with respect to glucose, the Pd-Ni composition provided better analytical characteristics, i.e., the lowest LOD, a higher sensitivity, and a broader linear concentration range. We suppose that this performance is due to the bimetallic composition of the electrode and a complex synergic electrocatalytic mechanism. Similar effects of the improved electrochemical sensor performance, e.g., significantly higher currents and as a consequence, a higher sensitivity of determination of hydrogen peroxide were observed for microwires of Au-Cu prepared by laser-induced metal deposition from solution in comparison with Au microwires (Panov et al., 2017). The enhanced electrocatalytic activity of two-component or more complex compositions of electrode materials in comparison with individual pure metal surfaces was also discussed (e.g., Koper, 2004; RodriGuez-Nieto et al., 2004; Shaidarova and Budnikov, 2011; Guascito et al., 2013; Yang et al., 2013). This effect may be due to bifunctional or synergic effect, the ligand or electronic effect with variations of electronic and catalytic properties of elements in composite materials compared with pure metals (Koper, 2004; RodriGuez-Nieto et al., 2004; Shaidarova and Budnikov, 2011), and formation of various phases and crystal defects in multicomponent systems leading to more electrocatalytically active surfaces. However, molecular understanding of bimetallic electrocatalysis and explanation of the experimentally observed enhanced electrocatalytic performance of bimetallic and multicomponent systems in comparison with individual metal surfaces require further detailed investigations in each case (Koper, 2004).

### Ethanol detection

Further characterization of the NW electrodes prepared by DENA included the study of their electrocatalytic performance in the oxidation of ethanol. Ethanol electrooxidation on metal and composite electrodes has been discussed in a number of reviews and articles (Azevedo et al., 2005; Chen and Ostrom, 2015; Liu et al., 2015; Shishov et al., 2016; Cinti et al., 2017). Ethanol electrooxidation was reported to be most effective on the Pd-containing electrodes in alkaline media due to the electrocatalytic activity of Pd (Liu et al., 2007; Ksar et al., 2009; Chen and Ostrom, 2015). As it was discussed above, metal oxy-species on the electrode surface are supposed to mediate the electrooxidation of ethanol on Pd electrodes. It was shown that Pd had no activity for ethanol oxidation in acid media (Liu et al., 2007). Therefore, in the following, results for the Pd-containing NW electrodes prepared by DENA in 0.1 M KOH solution are presented. The oxidation sequence for ethanol oxidation in alkaline media may be summarized as follows:

(11) $$\begin{array}{l} \text{O}{\text{H}}^{-}\to \text{O}{\text{H}}_{\text{ads}}+e^{-} \end{array}$$

(12) $$\begin{array}{l} \text{C}{\text{H}}_{3}\text{C}{\text{H}}_{2}\text{OH}+3\text{O}{\text{H}}^{-}\to \text{C}{\text{H}}_{3}\text{C}{\text{O}}_{\text{ads}}+3{\text{H}}_{2}\text{O}+3e^{-} \end{array}$$

(13) $$\begin{array}{l} \text{C}{\text{H}}_{3}\text{C}{\text{O}}_{\text{ads}}+\text{O}{\text{H}}_{\text{ads}}\to \text{C}{\text{H}}_{3}\text{COOH} \end{array}$$

(14) $$\begin{array}{l} \text{C}{\text{H}}_{3}\text{COOH}+\text{O}{\text{H}}^{-}\to \text{C}{\text{H}}_{3}\text{CO}{\text{O}}^{-}+{\text{H}}_{2}\text{O} \end{array}$$

**Figure 8:**
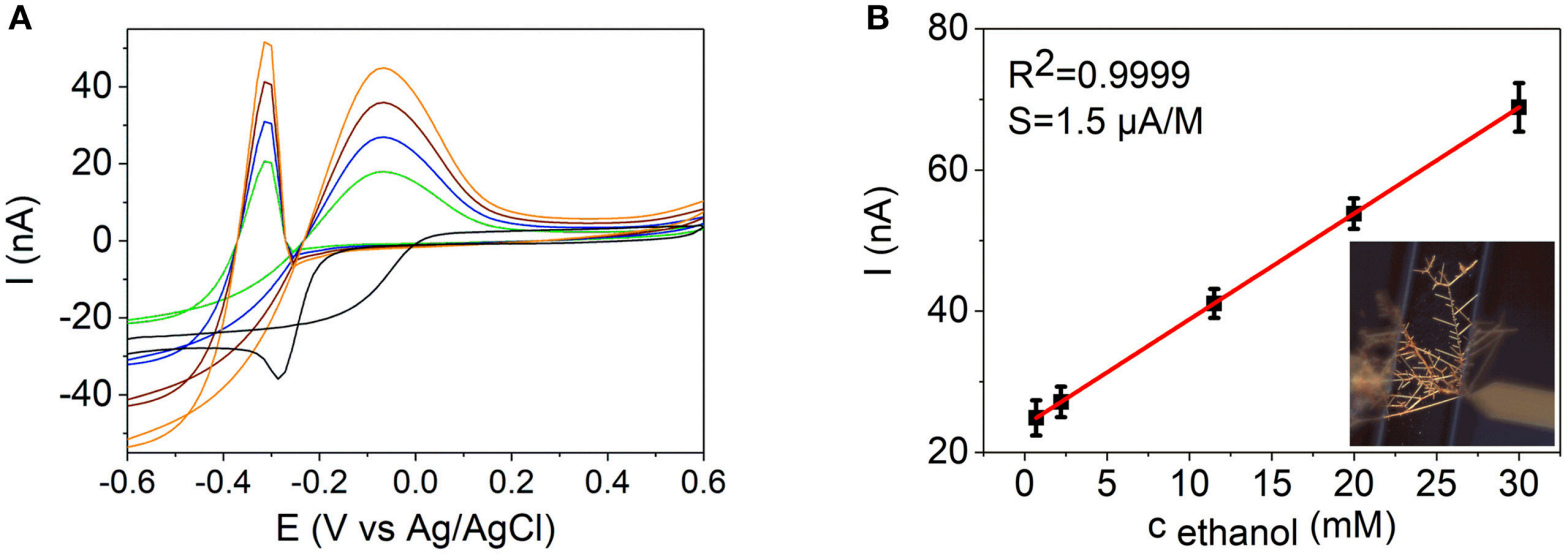
**(A)** Cyclic voltammograms of a Pd NW electrode in a 0.1 M KOH (black line) with additions of ethanol up to 3.0 × 10^−2^ M. **(B)** Dependence of the anodic current on the concentration of ethanol at −0.3 V in 0.1 M KOH at the Pd NW electrode. Insert in **(B)** shows the Pd nanodendrite electrode used for the detection.

**Figure 9:**
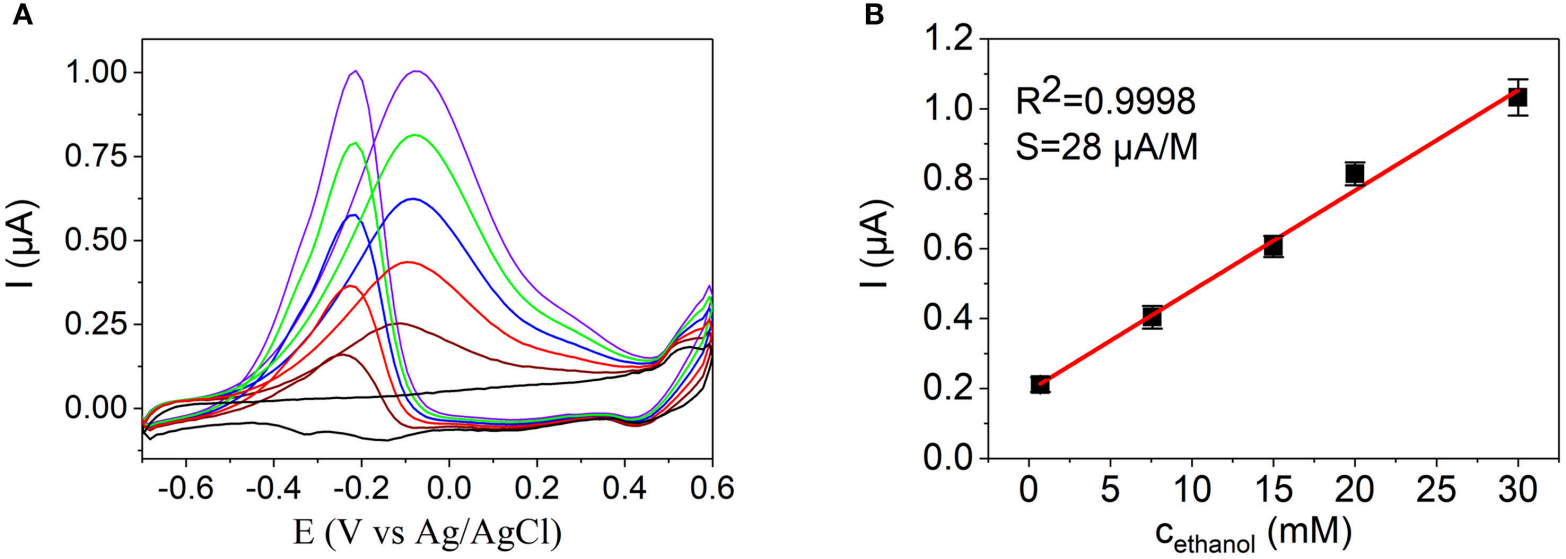
**(A)** Cyclic voltammograms of a Pd-Ni NW electrode in a 0.1 M KOH (black line) with additions of ethanol up to 3.0 × 10^−2^ M. **(B)** Calibration curve of the Pd-Ni NW sensor as a dependence of the anodic current on the concentration of ethanol at −0.25 V in 0.1 M KOH.

Figures 8, 9 show voltammograms of the Pd- and Pd-Ni NW electrodes prepared by DENA in 0.1 M KOH without and with successive additions of ethanol. The current-voltage curves in the presence of ethanol display two well-defined anodic current peaks: one on the forward (anodic) potential scan and another one on the reverse (cathodic) sweeping (Gutiérrez et al., 2007; Ksar et al., 2009), Figures 8A, 9A. The appearance of a symmetric anodic peak in the forward scan at about −0.070 V corresponds to the ethanol electrooxidation process. In the reverse scan, oxidation of the incompletely oxidized and adsorbed on the electrode surface intermediate carbonaceous species, which are produced in the forward anodic scan, results in the appearance of the asymmetric anodic peak at about −0.300 V for Pd- and −0.230 V for Pd-Ni NW electrodes.

The characteristics of the NW sensors for ethanol detection based on the electrocatalytic oxidation of ethanol are presented in Table 3 and Figures 8, 9. A linear calibration range of 7.0 × 10^−4^ − 3.0 × 10^−2^ M (*R*^2^ = 0.9999), detection limit of 2.2 × 10^−4^ M (*S/N* = 3), and a sensitivity of 1.5 μA M^−1^ were found for the Pd NW electrodes. For the Pd-Ni NW electrodes, a similar linear calibration range of 7.0 × 10^−4^ − 3.0 × 10^−2^ M (*R*^2^ = 0.9998) with a detection limit of 5.2 × 10^−4^ M, and a sensitivity of 28 μA M^−1^ were observed in alkaline media.

### Hydrogen peroxide detection

Further characterization of the metal NW electrodes prepared by DENA included the study of their performance in the reduction of hydrogen peroxide, thereby Pt- and Pd-based electrocatalysts generally demonstrate high catalytic activity for the electrochemical reduction of hydrogen peroxide (Chen and Ostrom, 2015). Non-enzymatic hydrogen peroxide electrochemical sensors have been discussed in a number of recent reviews and articles (Chen et al., 2014; Chen and Ostrom, 2015; Naveen et al., 2016; Plauck et al., 2016; Wang et al., 2016; Komkova et al., 2017; Nikolaev et al., 2018).

**Figure 10:**
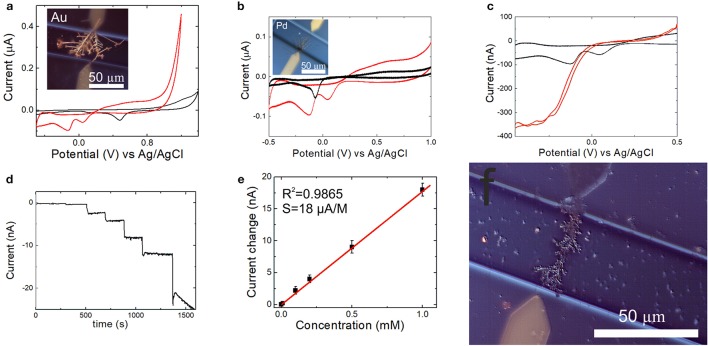
**(a)** Cyclic voltammogramms of the Pd-Au (red line) and Au (black line) NW electrodes assembled by DENA in 0.1 M PBS, pH 7.2. **(b)** CVs of the Pd-Au (red line) and Pd (black line) NW electrodes in 0.1 M PBS, pH 7.2. **(c)** CVs of the Pd-Au NW electrode at 0 mM (black line) and 10 mM H_2_O_2_ (red line), 0.1 M PBS, pH 7.2. **(d)** Amperogram with additions of H_2_O_2_ in 0.1 M PBS, pH 7.2, at E_det_ = −0.05 V. H_2_O_2_ concentration in the final solution was 10^−3^ M. **(e)** Calibration curve of the sensor in 0.1 M PBS, pH 7.2, at E_det_ = −0.05 V. **(f)** Optical microscopy image of the Pd-Au NW electrode assembled by DENA and used for the electrochemical measurements. Reprinted by permission from: Springer, J. Solid State Electrochemistry, Bimetallic nanowire sensors for extracellular electrochemical hydrogen peroxide detection in HL-1 cell culture, Konstantin G. Nikolaev, $Vanessa Maybeck, Elmar Neumann, Sergey S. Ermakov, Yury E. Ermolenko, Andreas Offenhäusser, Yulia G. Mourzina (2017), advance online publication, 28.11.2017 (doi: 10.1007/s10008-017-3829-3).

Electrochemical reduction of hydrogen peroxide on several nanodendrite electrode surfaces was studied to evaluate these electrode materials with regard to their application in hydrogen peroxide sensing. The electrochemical measurements were performed at pH 7.2 maintained with 0.1 M PBS, Figure 10. The current-voltage curves, which were recorded in 0 mM (black line) and 10 mM (red line) solutions of hydrogen peroxide on the Pd-Au nanodendrite electrode, and the corresponding electrode are shown in Figures 10c,f, respectively. As can be seen, the Pd-Au electrode demonstrates high electrochemical activity toward hydrogen peroxide reduction at these conditions with a half-wave reduction potential of about −0.125 V (vs. Ag/AgCl). Additionally, analytical characteristics of the sensors in terms of linear concentration range, sensitivity, and detection limit were studied by amperometry, where a detection potential of as low as −0.05 V was selected, Figures 10d,e. A low absolute value of the detection potential for the amperometric measurements was used to show the feasibility of the sensor application for the analysis of reactive oxygen species and oxygen metabolism in biological systems (Mason, 1957; Calas-Blanchard et al., 2014). The Pd-Au NW sensor demonstrates a high sensitivity of 18 μA M^−1^ in a wide linear concentration range of 10^−6^ − 10^−3^ M of hydrogen peroxide at this detection potential, Table 4. Selectivity of the sensor response to a number of interfering substances was evaluated, Figure S2. The response of the sensor to 1 μM hydrogen peroxide decreased to < 50% in the presence of 1.5 × 10^−4^ M ascorbate, 1 × 10^−4^ M dopamine, and 5 × 10^−4^ M uric acid. Thus, our results show that high concentrations of these substances exhibit interfering effect on the determination of hydrogen peroxide, however, do not distort the response to hydrogen peroxide essentially, which allows to use the developed sensors and multisensor systems in the presence of these interfering substances. The interfering effect is due to the redox behavior of the substances on electrodes and reduction of hydrogen peroxide by these interfering substances (Ames et al., 1981; Lowry and O'Neill, 1992; Deutsch, 1998; Zhao and Kim, 2016). Similar effects were discussed in Lowry and O'Neill (1992).

**Table 4:** Analytical characteristics of the detection of hydrogen peroxide in solutions with Au-, Pd-, and Pd-Au nanodendrite electrodes electrodeposited by DENA, 0.1 M PBS.

**Metal/alloy**	**Linear range, M**	**LOD, M**	**Sensitivity, A M^−1^**
Au	2 × 10^−4^ − 1 × 10^−2^	5.9 × 10^−5^	5.7 × 10^−7^
Pd	1 × 10^−5^ − 7.9 × 10^−3^	6.0 × 10^−6^	5.4 × 10^−7^
Pd-Au	1 × 10^−6^ − 1 × 10^−3^	2.4 × 10^−7^	1.8 × 10^−5^

Table 4 summarizes the analytical characteristics of the Pd-Au, Au, and Pd nanodendrite electrodes assembled by DENA. It can be seen, that the Pd-Au NW electrodes show a wide linear range with higher sensitivity, and a lower detection limit in comparison with the Au and Pd NW electrodes. Essentially, these analytical characteristics were achieved at a significantly lower absolute value of the detection potential than in a series of previous works (Chen et al., 2013, 2014; Goran et al., 2015; Huang et al., 2015), thus emphasizing high activity of these new sensor nanomaterials toward electrochemical reduction of H_2_O_2_.

Analytical performance of the DENA-prepared non-enzymatic electrochemical sensors in electrolyte solutions offer wide linear concentrations intervals, low detection limits, and additional benefits resulting from the multiplicity of possible material compositions, spatial resolution, and durability of the sensors. While fabrication of enzymatic and most non-enzymatic sensors employs drop-casting of the nanostructures prepared by different methods and other onerous immobilization procedures on the glassy carbon electrodes or other supports, the DENA sensors presented in this investigation do not involve modification of the electrode surfaces as well as the sensor response is not affected by the surface protection (surface-capping) agents from the chemical synthesis of the nanostructures. Hence, the sensors are stable to the detachment of components, which makes them favorable for *in vivo* analysis, minimizes drift of the sensor response, and thereby improves the stability. Furthermore, significantly lower absolute values of the detection potentials than in many previous investigations (Azevedo et al., 2005; Chen et al., 2013, 2014; Goran et al., 2015; Huang et al., 2015; Jin et al., 2015; Pourbeyram and Mehdizadeh, 2016; Shishov et al., 2016; Shabnam et al., 2017) demonstrate that the DENA-metal NW electrodes possess high activity for the electrochemical redox processes of a number of substances such as glucose, ethanol, and hydrogen peroxide, which are both important analytes and components of the fuel cell electrochemistry. Possible approaches to compensate for a generally lower selectivity of the non-enzymatic design in comparison with enzymatic biosensors can be involvement of the separation methods as well as realization of the voltamperometric “electronic tongue”-type multisensor systems, where non-selective signals of multisensor arrays are processed by chemometric techniques.

## Conclusions

Advances in nanotechnology, nanomaterials, and chemometric methods provide new opportunities for innovative electrochemical non-enzymatic sensors, multisensor systems, and multicomponent analysis. In this paper, we discuss DENA of nanowire electrochemical sensors and sensor arrays of metals and bimetallic compositions for the development of a single-chip multisensor system for the solution analysis. Various sensor nanomaterials (Pd, Ni, Au, and their multicomponent compositions) were electrochemically assembled on a single chip and at the same time connected to the external circuit without employing repetitive cycles of photolithography. The structural features of the DENA-assembled electrodes were of 50 nm to several μm in diameter as found by SEM analysis. The nanostructures were characterized by EDX analysis with elemental mapping to confirm the presence of elements. Individual amperometric signals of the DENA-assembled NW electrodes of different compositions were analyzed to make use of the specific electrochemical surface properties and show their successful application as functional sensor devices. Characteristics of non-enzymatic electrooxidation or electroreduction of analytes (glucose, ethanol, and hydrogen peroide) varied significantly depending on the NW electrode composition. For example, Pd-Ni nanowire sensors based on non-enzymatic glucose oxidation were characterized by a linear concentration range of 1.5 × 10^−7^ − 2.0 × 10^−3^ M glucose (*R*^2^ = 0.9999) with a LOD of 4.0 × 10^−8^ M, and a sensitivity of 178 μA M^−1^ at a low value of the detection potential of −0.15 V. Pd- and Pd-Ni NW sensors demonstrated a similar linear calibration range of 7.0 × 10^−4^ − 3.0 × 10^−2^ M for ethanol determination with a higher sensitivity of 28 μA M^−1^ at −0.25 V for the Pd-Ni nanodendrite electrode. Pd-Au nanowire sensors based on the non-enzymatic hydrogen peroxide reduction demonstrated a linear concentration range of 10^−6^ − 10^−3^ M H_2_O_2_ with a LOD of 3 × 10^−7^ M, and a sensitivity of 18 μA M^−1^ at a low absolute value of the detection potential of −0.05 V. Thus, a novel single-chip electrochemical multisensor platform can be proposed based on the DENA-metal nanowire electrodes of a compositional diversity. We anticipate that DENA-nanomaterials will find a wide range of applications as electrochemical sensors and multisensor systems in the fields of high-resolution multicomponent monitoring in fundamental biology, pharmacology, biomedicine, catalysis, and microfuel cells to realize the synergetic effects of electrocatalytic materials. Possible approach to compensate for a generally lower selectivity of the non-enzymatic design in comparison with enzymatic biosensors can be realization of the voltamperometric electronic tongue-type multisensor systems, where non-selective signals of multisensor arrays are processed by chemometric techniques.

## Author contributions

KN made substantial contributions to design, acquisition, analysis, and interpretation of data, and participated in drafting the article. YE and SE made substantial contributions to conception, analysis, and interpretation of data, and participated in drafting the article. AO made substantial contributions to conception and interpretation of data, and participated in drafting the article. YM made substantial contributions to conception, design, acquisition, analysis, and interpretation of data, and participated in drafting the article. All authors gave final approval of the submitted manuscript.

### Conflict of interest statement

The authors declare that the research was conducted in the absence of any commercial or financial relationships that could be construed as a potential conflict of interest.
