# Supplementary material for: Multisensor Systems by Electrochemical Nanowire Assembly for the Analysis of Aqueous Solutions
Source: Front Chem. 2018 Jun 29;6:256. doi: 10.3389/fchem.2018.00256 (PMC6034576; doi:10.3389/fchem.2018.00256)
Supplement: Supplementary file 1 [file Data_Sheet_1.pdf]

Supporting Information

Video S1

Video of the DENA processes of a Pd-Au nanodendrite electrode from a water solution of 5×10^{-3} M K_2PdCl_4 and 5×10^{-3} M $HAuCl_4$ (Leica optical microscope).

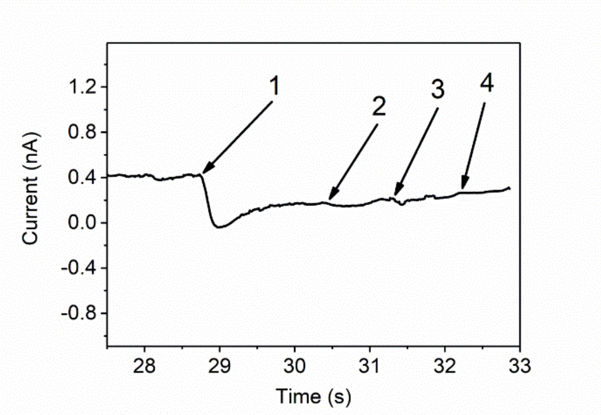

Figure S1 | Response of an Au NW sensor to 1 - 0.01 M glucose and in the presence of 2 - 10^{-3} M sucrose, 3 - 10^{-3} M fructose, and 4 - 10^{-4} M ascorbic acid.

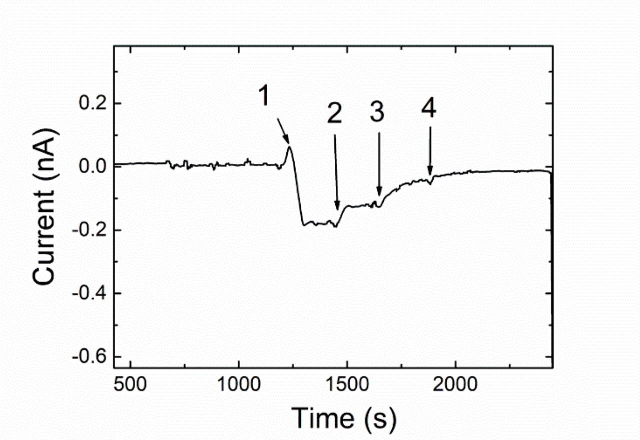

Figure S2 | Response of a Pd-Au NW sensor to 1 - 1 µM H_2O_2 and in the presence of 2 - 10^{-3} M dopamine, 3 - 0.5×10^{-3} M uric acid, and 4 - 0.15×10^{-3} M ascorbic acid.
